# A Method to screen U.S. environmental biomonitoring data for race/ethnicity and income-related disparity

**DOI:** 10.1186/1476-069X-12-114

**Published:** 2013-12-19

**Authors:** Anna Belova, Susan L Greco, Anne M Riederer, Lauren E W Olsho, Mark A Corrales

**Affiliations:** 1 Abt Associates, 4550 Montgomery Avenue, Suite 800 North, 20814 Bethesda, MD, USA; 2 Department of Environmental Health, Emory University, 1518 Clifton Rd NE, 30322 Atlanta, GA, USA; 3 Abt Associates, 55 Wheeler Street, 02138 Cambridge, MA, USA; 4 Environmental Protection Agency, 1200 Pennsylvania Avenue NW, 20460 Washington, DC, USA

**Keywords:** Environmental justice, Race, Ethnicity, Income, Metals, Dichlorophenol, Phthalates, NHANES, Biomonitoring

## Abstract

**Background:**

Environmental biomonitoring data provide one way to examine race/ethnicity and income-related exposure disparity and identify potential environmental justice concerns.

**Methods:**

We screened U.S. National Health and Nutrition Examination Survey (NHANES) 2001–2008 biomonitoring data for 228 chemicals for race/ethnicity and income-related disparity. We defined six subgroups by race/ethnicity—Mexican American, non-Hispanic black, non-Hispanic white—and income—Low Income: poverty income ratio (PIR) <2, High Income: PIR ≥ 2. We assessed disparity by comparing the central tendency (geometric mean [GM]) of the biomonitoring concentrations of each subgroup to that of the reference subgroup (non-Hispanic white/High Income), adjusting for multiple comparisons using the Holm-Bonferroni procedure.

**Results:**

There were sufficient data to estimate at least one geometric mean ratio (GMR) for 108 chemicals; 37 had at least one GMR statistically different from one. There was evidence of potential environmental justice concern (GMR significantly >1) for 12 chemicals: cotinine; antimony; lead; thallium; 2,4- and 2,5-dichlorophenol; p,p’-dichlorodiphenyldichloroethylene; methyl and propyl paraben; and mono-ethyl, mono-isobutyl, and mono-n-butyl phthalate. There was also evidence of GMR significantly <1 for 25 chemicals (of which 17 were polychlorinated biphenyls).

**Conclusions:**

Although many of our results were consistent with the U.S. literature, findings relevant to environmental justice were novel for dichlorophenols and some metals.

## Background

Environmental justice (EJ) concerns can arise when racial/ethnic minorities or those with lower socioeconomic status (SES) experience greater exposures to environmental pollutants than the rest of the population. Demographic variables used to characterize SES can include income, education, or occupation. Many EJ studies have focused on disparities in exposure to ambient air pollutant levels. Studies on hazardous air pollutants have found higher cancer risks associated with lower SES, higher proportion of African Americans, and lower proportion of whites in a census tract [1]; higher level of racial segregation [2]; and higher proportion of Hispanics [3]. U.S. counties with the highest fine particulate matter (PM_2.5_) and ozone levels had higher percentages of people living in poverty and non-Hispanic black residents [4], and Hispanics and non-Hispanic blacks had higher exposures to PM_2.5_ components than whites [5].

Environmental biomonitoring—assessing exposure to pollutants/chemicals by measuring them or their metabolites in blood, urine, or other specimens—provides a complementary approach to examining potential disparities and identifying EJ concerns. Biomonitoring concentrations (i.e., biomarkers) reflect the amount of chemical entering the body from all sources (air, water, food, soil, dust, consumer products) via all exposure routes (ingestion, inhalation, dermal absorption) [6]. One chemical may be assessed in the body using several biomarkers (e.g., lead in blood and urine). Biomarkers are particularly informative when source- and route-specific data are limited. However, detailed studies are required to link biomarker concentrations back to environmental exposures for policy-setting purposes. Biomarkers also reflect how a given individual absorbs, distributes, metabolizes, and excretes the chemical (i.e., toxicokinetics), all of which may be influenced by genetic and epigenetic characteristics that could vary by race/ethnicity or SES [7,8]. Furthermore, the presence of an environmental chemical in an individual’s blood or urine does not imply that this chemical causes disease [6].

To date, most detailed studies of race/ethnicity or income-related disparities using biomarker data have been hypothesis-driven, focusing on a few chemicals selected based on known or suspected exposure disparities and controlling for relevant covariates. This approach may miss important disparities in exposure to less studied chemicals. A screening-level analysis of a large number of biomarkers for differential exposure could identify additional candidates for detailed study of the potential magnitude, drivers, and public health relevance of any race/ethnicity or income-related disparities.

The U.S. Centers for Disease Control and Prevention (CDC) collects and tracks environmental biomonitoring data through the National Health and Nutrition Examination Survey (NHANES). The *Fourth National Report on Human Exposure to Environmental Chemicals*[6] examines concentrations of 212 chemicals in NHANES 1999–2004, providing means and select percentiles stratified by survey years, age group, sex, and race/ethnicity. (Tables were recently updated for 117 chemicals, and they incorporate 34 new chemicals from NHANES 2005–2010 [9].) However, the report does not statistically compare biomarkers across racial/ethnic subgroups, or consider income, an important EJ dimension.

One example of an exploratory assessment for a large number of chemicals is a recent study by Tyrrell et al. that investigated associations between income and levels of 179 chemicals in NHANES 2001–2010 [10]. The authors used linear regression modeling to test for significant associations between the poverty income ratio (PIR) and log-transformed biomarker concentrations, controlling for age, sex, race, and waist circumference. For chemicals with significant negative PIR associations in at least two NHANES cycles, Tyrrell et al. used structural equation modeling to explore the pathways through which income impacts the biomarker concentrations. However, Tyrrell et al. did not use a formal procedure to adjust for multiple testing, implying that some of their significant findings could be spurious.

To demonstrate a formal screening method, we analyzed all biomarkers in the NHANES 2001–2008 datasets for differences in concentration across U.S. population subgroups defined by race/ethnicity and income. We build upon the *Fourth Exposure Report* and on Tyrrell et al. [10] by: (1) modeling joint impacts of race/ethnicity and income; (2) testing for statistically significant evidence of disparity with proper adjustments for multiple comparisons; and (3) addressing measurements below the limit of detection (LOD) using variable-threshold censored regression. This screening method focuses on differences in mean biomarker concentrations among subgroups.

## Methods

### Data

NHANES collects nationally representative environmental biomonitoring data from approximately 2,500 participants in each two-year cycle [6]. Ethical approval for use of NHANES data that is freely available on the web is not required as it is anonymized. We analyzed data from 19 of the most recently available (as of May 2011) NHANES laboratory and demographic files, corresponding to 2001–2002 (1 chemical), 2003–2004 (162 chemicals), and 2007–2008 (65 chemicals) [11-14]. We aggregated chemicals into 10 groups: cotinine, halogenated aromatics, metals, polycyclic aromatic hydrocarbons (PAHs), polyfluoralkyl chemicals (PFCs), perchlorate, pesticides, phenols, phthalates, and volatile organic compounds (VOCs) [see Table A1 in Additional file 1]. Each chemical could be measured in different media and/or using different corrections; we defined these different measures as separate biomarkers. For example, the chemical lead (Pb) was measured in blood and urine, reported as both unadjusted and creatinine-corrected. Thus, there were three biomarkers associated with the chemical Pb. We analyzed a total of 410 biomarkers corresponding to 228 chemicals. We parallel the presentation of units in the *Fourth Exposure Report*[6]. We present urinary concentrations per volume of urine and per gram of creatinine. While creatinine correction should account for urine dilution in spot urine samples, creatinine levels can vary by age, sex, race, renal function, lean muscle mass, and red meat consumption [15]. Lipophilic chemicals (such as dioxins, furans, and polychlorinated biphenyls [PCBs]) are presented per gram of total lipid (reflecting the amount stored in body fat) as well as per whole weight of serum. Other chemicals measured in serum are presented per liter of serum. For each biomarker, we calculated the LOD by multiplying reported concentrations by √2 for observations flagged “below LOD.”

We computed the relevant summary measures for Mexican American, non-Hispanic black, and non-Hispanic white race/ethnicity categories available in NHANES, but not the other Hispanic or “other race” categories because their small sample sizes do not permit generating reliable estimates [16] and because of potential heterogeneity of exposure patterns in these subpopulations [17]. To categorize participants by income, we used the PIR reported by NHANES. PIR is a family’s total income divided by the family size-specific poverty threshold income, which is published in the *Federal Register* by the U.S. Department of Health and Human Services. While some NHANES studies used a three-way PIR-based classification, e.g., *poor* (PIR < 1), *near poor* (1 ≤ PIR < 2), and *not poor* (PIR ≥ 2) [18,19], we found that a three-way PIR-based categorization often results in small subgroup sample sizes when combined with a three-way race/ethnicity-based categorization. Instead, we employed a pseudo-balanced two-way categorization (since the unweighted median PIR in our dataset was close to 2), defining “Low” Income (PIR < 2) and “High” Income (PIR ≥ 2) subgroups. A PIR threshold of 2 is used by some U.S. agencies as a qualifier for government assistance [20] and was also used to explore Vitamin D deficiency using NHANES data [21].

Thus, we classified individuals into six race/ethnicity and income subgroups, with non-Hispanic white/High Income serving as the reference subgroup. For each biomarker, we analyzed data for all participants with non-missing biomarker measurements and PIR. There were no individuals with missing race/ethnicity status in the NHANES datasets we examined. Depending on the biomarker, the final analytic sample included between 90% and 95% of all participants with non-missing biomarker measurements.

### Analysis

Following CDC [6], we assumed that biomarker concentrations could be treated as lognormally distributed, and used the geometric mean (GM) as the measure of central tendency. Biomarker concentrations were censored by the LOD, which could be individual-specific for some biomarkers. While replacement of concentrations below the LOD by LOD/√2 has been employed [6], this type of substitution has been shown to generate biased estimates [22,23]. In our analysis, we accommodated the LOD censoring by estimating variable-threshold censored regression models [22,24].

Specifically, for each biomarker *b* we evaluated the following pseudo-log-likelihood function ln *L*_*b*_:

(1) $$\begin{array}{ll} \;\ln L_{b}= & \sum_{c_{\mathrm{bi}}\leq \mathrm{LO}D_{\mathrm{bi}}}w_{\mathrm{bi}}\ln\Phi \left(\frac{\ln{\mathrm{LOD}}_{\mathrm{bi}}-\mu_{\mathrm{bi}}}{\sigma_{\mathrm{bi}}}\right)\\ & +\sum_{c_{\mathrm{bi}}>\mathrm{LO}D_{\mathrm{bi}}}w_{\mathrm{bi}}\ln\left(\frac{1}{c_{\mathrm{bi}}\sigma_{\mathrm{bi}}}\phi \left(\frac{\ln c_{\mathrm{bi}}-\mu_{\mathrm{bi}}}{\sigma_{\mathrm{bi}}}\right)\right), \end{array}$$

where *c*_*bi*_ is the concentration of biomarker *b* measured in the *i*th individual, LOD_*bi*_ is the LOD for that biomarker for the *i*th individual; *w*_*bi*_ is the individual-specific survey weight; Φ(.) is the cumulative standard normal distribution and *ϕ*(.) is the standard normal distribution; and *μ*_*bi*_ and *σ*_*bi*_ are the arithmetic mean and the arithmetic standard deviation of ln *c*_*bi*_ for the *i*th individual, respectively. We constrained *μ*_*bi*_ (and *σ*_*bi*_) to be the same for participants in the same subgroup *s*, permitting estimation of subgroup-specific GMs and geometric standard deviations (GSDs). In other words, we sought to maximize the pseudo-log-likelihood function in equation (1) under simple linear equality constraints. If ${\hat{\mu }}_{\mathrm{bs}}$ and ${\hat{\sigma }}_{\mathrm{bs}}$ denote the estimates of *μ*_*bs*_ and *σ*_*bs*_ for biomarker *b* in subgroup *s*, then the estimated GM and GSD for this biomarker in this subgroup are $\exp\left({\hat{\mu }}_{\mathrm{bs}}\right)$ and $\exp\left({\hat{\sigma }}_{\mathrm{bs}}\right),$ respectively.

We followed CDC’s convention of not reporting GM estimates for subsamples with >40% of results below the LOD [6]. We estimated sampling variances of parameters in the constrained version of the model in equation (1) using the Taylor series method [25], which relies on results in Binder [26]. Methods described in [27] were used to generate point and range estimates for subpopulations of interest. Specifically, to accommodate the complex design and laboratory subsample weights in our subpopulation analysis, we employed Stata/SE 11.2 [28,29] < svy, subpop(if …): intreg > programming statements. When laboratory weights were not provided, we followed CDC/NCHS [25] and used the two-year examination weights. Weighted NHANES estimates are representative of the U.S. civilian, non-institutionalized population.

### Testing for disparity

We assessed potential race/ethnicity and income-related disparity at the center of each biomarker distribution using the following metric:

(2) $${\mathrm{GMR}}_{\mathrm{bs}}=\frac{{\mathrm{GM}}_{\mathrm{bs}}}{{\mathrm{GM}}_{\mathrm{br}}},$$

where GMR_*bs*_ is the ratio of the GM of biomarker *b* in subgroup s (GM_*bs*_) with respect to the GM of biomarker *b* in the reference subgroup *r* (GM_*br*_). Non-Hispanic white/High Income was the reference subgroup. Up to five GM comparisons could be made for each biomarker.

A particular subgroup-specific GM_*bs*_ is not different from the reference subgroup GM_*br*_ when GMR_*bs*_ = 1. For each biomarker *b* and subgroup s, we tested the null hypothesis that GMR_*bs*_ = 1 using two-sided tests because we had no *a priori* beliefs about directionality.

Because GMR involves a non-linear transformation of equation (1) parameters, whose estimators are t-distributed, the sampling distribution of the GMR estimator $\exp\left({\hat{\mu }}_{\mathrm{bs}}-{\hat{\mu }}_{\mathrm{br}}\right)$ is not known. Therefore, the tests were carried out in the log-space, by evaluating the hypothesis *μ*_*bs*_ - *μ*_*br*_ = 0 rather than GMR_*bs*_ = 1. Along with estimates ${\hat{\mu }}_{\mathrm{bs}}$ and ${\hat{\mu }}_{\mathrm{br}}$, Stata/SE 11.2 reports survey design-adjusted estimates of the relevant variances— $\hat{V}\left(\mu_{\mathrm{bs}}\right)$, $\hat{V}\left(\mu_{\mathrm{br}}\right)$ —and covariances— $\hat{C}\left(\mu_{\mathrm{bs}},\mu_{\mathrm{br}}\right)$. Under the null hypothesis, the sampling distribution of $\left({\hat{\mu }}_{\mathrm{bs}}-{\hat{\mu }}_{\mathrm{br}}\right)/\sqrt{\hat{V}\left(\mu_{\mathrm{bs}}\right)+\hat{V}\left(\mu_{\mathrm{br}}\right)-2\hat{C}\left(\mu_{\mathrm{bs}},\mu_{\mathrm{br}}\right)}$ is a central t-distribution with degrees of freedom determined by the NHANES survey design features. This distribution was used to derive p-values for each test. The confidence intervals for the GMRs were obtained by exponentiating the confidence intervals for *μ*_*bs*_ - *μ*_*br*_.

Our screening analysis involved multiple testing of the hypothesis GMR_*bs*_ = 1 for several subgroup-specific GMRs and a large number of biomarkers. A large number of false positives is expected with this many comparisons. Therefore, we capped the probability of encountering at least one false positive among all tests at 0.05 using the Holm-Bonferroni procedure [30]. That is, we controlled the family-wise error rate (FWER) at 5%, where the family of tests was the entire collection of comparisons. This allowed us to summarize the screening results for all biomarkers and subgroups together [31]. This approach follows best practices in biomedical research and conforms to the guidelines of the U.S. Food and Drug Administration, which recommends controlling the FWER in clinical trials [32]. Last, we qualitatively validated our statistically significant results by reviewing the published literature on those biomarkers for evidence of disparity.

## Results

Although we examined 228 chemicals, there were only 108 chemicals for which at least one GMR could be estimated. Among the 795 GM comparisons across subgroups and biomarkers, there were 37 chemicals with significant evidence of disparity: 12 chemicals with at least one GMR significantly >1, indicating potential EJ concerns, and 25 chemicals with at least one GMR significantly <1, indicating higher exposures in the reference subgroup (non-Hispanic white/High Income). Additional information on the overall GMR screening results at the comparison, biomarker, and chemical level is provided in Table A2 [see Additional file 1].

**Figure 1:**
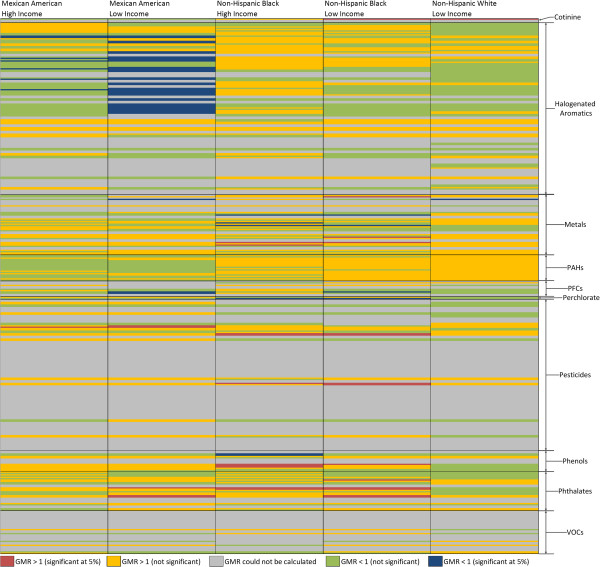
**Visual overview of GMR for NHANES environmental biomonitoring data for all subgroups.** Each cell of the matrix summarizes the outcome of the geometric mean ratio (GMR) test performed. The key to the color codes is located under the matrix. The columns correspond to the five race/ethnicity subgroups (Mexican American/High Income; Mexican American/Low Income; non-Hispanic black/High Income; non-Hispanic black/Low Income; non-Hispanic white/Low Income) that are being compared to the reference subgroup (non-Hispanic white/High Income). The rows of the matrix correspond to 410 studied biomarkers. The chemical groups to which these biomarkers belong (cotinine; halogenated aromatics; metals; polycyclic aromatic hydrocarbons, PAHs; polyfluoralkyl chemicals, PFCs; perchlorate; pesticides; phenols; phthalates; volatile organic compounds, VOCs) are indicated along the right edge of the matrix.

Figure 1 provides a visual overview of the results. A few broad patterns can be discerned. First, the predominance of grey indicates that many GMRs could not be calculated because of the large number of non-detectable concentrations. Second, the relatively small number of red and blue cells indicates that the GM concentrations in the subgroups were rarely significantly different from those of the reference subgroup for the biomarkers with computable GMRs. This could be due to the fact that the differences were not large or there was insufficient power to detect these differences. Third, there were instances where Mexican Americans, particularly low income, had significantly lower levels of biomarkers than the reference subgroup. Fourth, biomarker levels for low-income, non-Hispanic whites were generally similar to those for high-income, non-Hispanic whites (the reference subgroup). Finally, evidence of significant EJ disparity is generally seen in non-Hispanic blacks (low and high income) and low-income Mexican Americans.

Examining results at the chemical level, additional observations can be made. Pesticides, phthalates, and cotinine contained biomarkers for which all GMRs significantly different from one were also >1, indicating potential EJ concern. Conversely, halogenated aromatics (PCBs in this case), PFCs, and perchlorate included biomarkers for which GMRs significantly different from one were exclusively <1, indicating higher exposures in the reference subgroup. Mixed results (GMRs both significantly >1 and <1) were encountered among phenols and metals. No evidence of significant disparity was found for PAHs or VOCs. However, a large fraction of GMRs could not be estimated for VOCs, pesticides, or halogenated aromatics.

Table 1 presents information on the 12 chemicals corresponding to 31 GMRs significantly >1, indicating potential EJ concerns for the following chemical groups: cotinine, metals, pesticides, phenols, and phthalates. Of the 31 GMRs >1, there were 14 for the non-Hispanic black/Low Income, 10 for the non-Hispanic black/High Income, 5 for the Mexican American/Low Income, 1 for the Mexican American/High Income, and 1 for the non-Hispanic white/Low Income subgroups. Sample sizes were consistently smallest for the Mexican American/High Income subgroup. The GMRs in Table 1 range from 1.3 to 12, but should not be compared across biomarkers except with great caution because of the differences in variability of the concentration levels across biomarkers.

**Table 1:** Chemicals demonstrating EJ concern (GMR > 1 at 5% joint significance level)

**Chemical group (NHANES cycle)**^**a**^	**Chemical name (units, media)**	**Subgroup**^**b**^		**Subgroup number of observations (ND)**^**c**^	**Subgroup geometric mean (95% CI)**^**d**^	**Reference subgroup number of observations (ND)**^**c,e**^	**Reference subgroup geometric mean (95% CI)**^**d,e**^	**Geometric mean ratio (95% CI)**^**d**^
		**Race/Ethnicity**	**Income**					
Cotinine (2007–2008)	COTININE (ng/mL, serum)	NHB	Low	787 (43)	1.7 (1.2–2.3)	1,752 (390)	0.14 (0.074–0.25)	12 (6.1–25)
	COTININE (ng/mL, serum)	NHW	Low	1,342 (104)	1.3 (0.67–2.4)	1,752 (390)	0.14 (0.074–0.25)	9.4 (6.5–13)
Metals (2007–2008)	ANTIMONY (ug/L, urine)	NHB	High	284 (30)	0.082 (0.076–0.089)	581 (154)	0.052 (0.046–0.059)	1.6 (1.4–1.8)
	ANTIMONY (ug/L, urine)	NHB	Low	264 (19)	0.088 (0.078–0.098)	581 (154)	0.052 (0.046–0.059)	1.7 (1.4–2.0)
	LEAD (ug/dL, blood)	NHB	Low	872 (0)	1.5 (1.4–1.7)	1,819 (0)	1.2 (1.1–1.3)	1.3 (1.2–1.4)
	LEAD (ug/L, urine)	NHB	Low	264 (1)	0.67 (0.62–0.72)	581 (19)	0.43 (0.40–0.47)	1.6 (1.4–1.7)
	THALLIUM (ug/L, urine)	NHB	High	284 (1)	0.18 (0.17–0.2)	581 (5)	0.14 (0.13–0.15)	1.3 (1.2–1.4)
Pesticides (2003–2004, 2007–2008)	2,4-DICHLOROPHENOL (ug/L, urine)	NHB	High	258 (10)	1.8 (1.4–2.2)	588 (65)	0.79 (0.69–0.89)	2.3 (1.7–2.9)
	2,4-DICHLOROPHENOL (ug/L, urine)	NHB	Low	297 (14)	1.7 (1.4–2.2)	588 (65)	0.79 (0.69–0.89)	2.2 (1.8–2.8)
	2,4-DICHLOROPHENOL (ug/g, urine, creatinine-adj.)	NHB	Low	297 (14)	1.4 (1.1–1.7)	588 (65)	0.81 (0.72–0.91)	1.8 (1.4–2.2)
	2,5-DICHLOROPHENOL (ug/L, urine)	NHB	High	258 (0)	28 (21–38)	588 (11)	6.0 (4.7–7.6)	4.7 (3.2–7.0)
	2,5-DICHLOROPHENOL (ug/g, urine, creatinine-adj.)	NHB	High	258 (0)	21 (15–28)	588 (11)	6.3 (4.9–8.0)	3.3 (2.3–4.8)
	2,5-DICHLOROPHENOL (ug/L, urine)	NHB	Low	297 (2)	28 (19–41)	588 (11)	6.0 (4.7–7.6)	4.7 (3.3–6.5)
	2,5-DICHLOROPHENOL (ug/g, urine, creatinine-adj.)	NHB	Low	297 (2)	23 (16–33)	588 (11)	6.3 (4.9–8.0)	3.7 (2.6–5.2)
	P,P’-DDE (ng/g, serum, lipid-adj.)	MA	High	127 (0)	420 (320–570)	524 (1)	210 (170–250)	2.1 (1.6–2.7)
	P,P’-DDE (ng/g, serum)	MA	Low	312 (0)	2.7 (2.2-3.4)	524 (1)	1.3 (1.0-1.6)	2.1 (1.6–2.8)
	P,P’-DDE (ng/g, serum, lipid-adj.)	MA	Low	312 (0)	450 (360–560)	524 (1)	210 (170–250)	2.2 (1.7–2.8)
Phenols (2007–2008)	METHYL PARABEN (ng/mL, urine)	NHB	High	258 (0)	170 (150–200)	588 (1)	51 (41–64)	3.3 (2.6–4.2)
	METHYL PARABEN (ng/g, urine, creatinine-adj.)	NHB	High	258 (0)	130 (110–150)	588 (1)	54 (42–69)	2.3 (1.7–3.1)
	METHYL PARABEN (ng/mL, urine)	NHB	Low	297 (1)	140 (110–180)	588 (1)	51 (41–64)	2.7 (1.9–3.9)
	PROPYL PARABEN (ng/mL, urine)	NHB	High	258 (4)	24 (18–31)	588 (44)	6.6 (5.0–8.6)	3.6 (2.6–5.1)
Phthalates (2007–2008)	MONO-ETHYL PHTHALATE (ng/mL, urine)	MA	Low	306 (0)	180 (160–220)	588 (0)	120 (100–140)	1.5 (1.3–1.8)
	MONO-ETHYL PHTHALATE (ng/mL, urine)	NHB	High	258 (0)	280 (230–330)	588 (0)	120 (100–140)	2.3 (1.8–2.8)
	MONO-ETHYL PHTHALATE (ng/g, urine, creatinine-adj.)	NHB	High	258 (0)	200 (170–250)	588 (0)	130 (110–140)	1.6 (1.3–1.9)
	MONO-ETHYL PHTHALATE (ng/mL, urine)	NHB	Low	297 (0)	240 (210–280)	588 (0)	120 (100–140)	2.0 (1.6–2.5)
	MONO-ETHYL PHTHALATE (ng/g, urine, creatinine-adj.)	NHB	Low	297 (0)	200 (180–220)	588 (0)	130 (110–140)	1.6 (1.3–1.9)
	MONO-ISOBUTYL PHTHALATE (ng/mL, urine)	MA	Low	306 (1)	10 (8.8–12)	588 (20)	6.0 (5.5–6.6)	1.7 (1.4–2.0)
	MONO-ISOBUTYL PHTHALATE (ng/g, urine, creatinine-adj.)	MA	Low	306 (1)	10 (8.5–12)	588 (20)	6.3 (5.8–6.8)	1.6 (1.3–1.9)
	MONO-ISOBUTYL PHTHALATE (ng/mL, urine)	NHB	Low	297 (1)	11 (10–12)	588 (20)	6.0 (5.5–6.6)	1.9 (1.6–2.1)
	MONO-ISOBUTYL PHTHALATE (ng/g, urine, creatinine-adj.)	NHB	Low	297 (1)	9.2 (8.5–9.9)	588 (20)	6.3 (5.8–6.8)	1.5 (1.3–1.6)
	MONO-N-BUTYL PHTHALATE (ng/mL, urine)	NHB	Low	297 (2)	27 (24–30)	588 (10)	16 (15–19)	1.6 (1.4–2.0)

Notes: (a) Biomarkers were assigned to chemical groups on the basis of chemical consistency and groupings from the NHANES laboratory files. Biomonitoring data for the results in the table come from NHANES 2007–2008 for all chemical groups except pesticides, for which some of the data also come from NHANES 2003–2004. (b) MA – Mexican American, NHB – non-Hispanic black, NHW – non-Hispanic white; Low income corresponds to family PIR < 2, High income corresponds to family PIR ≥ 2 (PIR is the poverty income ratio). (c) The number of observations is unweighted. The number of non-detects (ND) is reported in parentheses. (d) The point estimate, followed by the range estimate in parentheses. The range estimate is a 95% confidence interval unadjusted for multiple comparisons. GM and GMR estimates are rounded to two significant figures. (e) The reference subgroup was non-Hispanic white/High income.

Table 2 presents information on the 25 chemicals corresponding to 55 GMRs significantly <1, indicating higher GMs in the non-Hispanic white/High Income reference subgroup. PCBs accounted for 17 of these 25. Of the 55 GMRs <1, most (41 PCB congeners) were among the halogenated aromatics, with others found among metals (7), perchlorate (2), PFCs (3), and phenols (2). For PCBs, many instances of GMR < 1 occurred for the Mexican American/Low Income subgroup.

**Table 2:** Chemicals with higher biomarker levels in the reference subgroup (GMR < 1 at 5% joint significance level)

**Chemical group (NHANES cycle)**^**a**^	**Chemical name (units, media)**	**Subgroup**^**b**^		**Subgroup number of observations (ND)**^**c**^	**Subgroup geometric mean (95% CI)**^**d**^	**Reference subgroup number of observations (ND)**^**c,e**^	**Reference subgroup geometric mean (95% CI)**^**d,e**^	**Geometric mean ratio (95% CI)**^**d**^
		**Race/Ethnicity**	**Income**					
Halogenated aromatics (2003–2004)	PCB74 (ng/g, serum)	MA	High	128 (0)	0.019 (0.016–0.023)	519 (0)	0.032 (0.03–0.035)	0.58 (0.48–0.72)
	PCB74 (ng/g, serum, lipid-adj.)	MA	High	128 (0)	3.0 (2.6–3.4)	519 (0)	5.3 (4.9–5.7)	0.57 (0.49–0.66)
	PCB74 (ng/g, serum)	MA	Low	276 (0)	0.013 (0.011–0.015)	519 (0)	0.032 (0.03–0.035)	0.40 (0.33–0.48)
	PCB74 (ng/g, serum, lipid-adj.)	MA	Low	276 (0)	2.1 (1.8–2.5)	519 (0)	5.3 (4.9–5.7)	0.41 (0.34–0.48)
	PCB99 (ng/g, serum)	MA	Low	275 (0)	0.013 (0.011–0.015)	517 (0)	0.026 (0.023–0.029)	0.51 (0.43–0.60)
	PCB99 (ng/g, serum, lipid-adj.)	MA	Low	275 (0)	2.2 (1.9–2.5)	517 (0)	4.2 (3.8–4.7)	0.52 (0.44–0.61)
	PCB118 (ng/g, serum)	MA	Low	275 (0)	0.019 (0.016–0.023)	516 (0)	0.037 (0.033–0.042)	0.51 (0.40–0.64)
	PCB118 (ng/g, serum, lipid-adj.)	MA	Low	275 (0)	3.2 (2.7–3.7)	516 (0)	6.1 (5.5–6.8)	0.52 (0.41–0.65)
	PCB138 & 158 (ng/g, serum)	MA	Low	276 (0)	0.037 (0.032–0.044)	517 (0)	0.097 (0.086–0.11)	0.38 (0.31–0.47)
	PCB138 & 158 (ng/g, serum, lipid-adj.)	MA	High	128 (0)	8.8 (7.3–11)	517 (0)	16 (14–18)	0.55 (0.44–0.69)
	PCB138 & 158 (ng/g, serum, lipid-adj.)	MA	Low	276 (0)	6.2 (5.3–7.2)	517 (0)	16 (14–18)	0.39 (0.32–0.48)
	PCB146 (ng/g, serum, lipid-adj.)	MA	High	127 (4)	1.1 (0.87–1.4)	519 (4)	2.3 (2.0–2.5)	0.48 (0.37–0.63)
	PCB146 (ng/g, serum)	MA	Low	275 (8)	0.0052 (0.0044–0.0061)	519 (4)	0.014 (0.012–0.016)	0.37 (0.3–0.46)
	PCB146 (ng/g, serum, lipid-adj.)	MA	Low	275 (8)	0.86 (0.73–1.0)	519 (4)	2.3 (2.0–2.5)	0.38 (0.31–0.47)
	PCB153 (ng/g, serum, lipid-adj.)	MA	High	127 (0)	11 (9.1–13)	519 (0)	22 (20–24)	0.50 (0.41–0.63)
	PCB153 (ng/g, serum)	MA	Low	275 (0)	0.046 (0.038–0.055)	519 (0)	0.13 (0.12–0.15)	0.35 (0.28–0.43)
	PCB153 (ng/g, serum, lipid-adj.)	MA	Low	275 (0)	7.7 (6.4–9.1)	519 (0)	22 (20–24)	0.36 (0.29–0.44)
	PCB156 (ng/g, serum)	MA	Low	273 (81)	0.0034 (0.002–0.0056)	515 (43)	0.019 (0.017–0.021)	0.18 (0.11–0.31)
	PCB156 (ng/g, serum, lipid-adj.)	MA	Low	273 (81)	0.58 (0.36–0.93)	515 (43)	3.1 (2.7–3.4)	0.19 (0.11–0.31)
	PCB170 (ng/g, serum, lipid-adj.)	MA	High	127 (6)	3.2 (2.6–4.0)	517 (5)	6.2 (5.8–6.7)	0.52 (0.41–0.65)
	PCB170 (ng/g, serum)	MA	Low	273 (17)	0.011 (0.0091–0.014)	517 (5)	0.038 (0.035–0.041)	0.30 (0.23–0.39)
	PCB170 (ng/g, serum, lipid-adj.)	MA	Low	273 (17)	1.9 (1.5–2.4)	517 (5)	6.2 (5.8-6.7)	0.31 (0.24–0.39)
	PCB177 (ng/g, serum)	MA	Low	273 (92)	0.0024 (0.0018–0.0033)	515 (67)	0.0069 (0.0062–0.0077)	0.35 (0.26–0.47)
	PCB177 (ng/g, serum, lipid-adj.)	MA	Low	273 (92)	0.41 (0.31–0.55)	515 (67)	1.1 (1.0–1.3)	0.36 (0.27–0.49)
	PCB180 (ng/g, serum, lipid-adj.)	MA	High	128 (1)	8.2 (6.3–11)	517 (2)	17 (16–19)	0.48 (0.36–0.62)
	PCB180 (ng/g, serum)	MA	Low	276 (4)	0.031 (0.024–0.039)	517 (2)	0.11 (0.096–0.12)	0.29 (0.23–0.38)
	PCB180 (ng/g, serum, lipid-adj.)	MA	Low	276 (4)	5.2 (4.1–6.4)	517 (2)	17 (16–19)	0.30 (0.24–0.38)
	PCB183 (ng/g, serum)	MA	Low	274 (45)	0.0036 (0.0029–0.0044)	518 (35)	0.0097 (0.0089–0.011)	0.37 (0.30–0.46)
	PCB183 (ng/g, serum, lipid-adj.)	MA	Low	274 (45)	0.60 (0.50–0.72)	518 (35)	1.6 (1.5–1.7)	0.38 (0.31–0.46)
	PCB187 (ng/g, serum)	MA	Low	275 (8)	0.0093 (0.0073–0.012)	519 (8)	0.028 (0.025–0.031)	0.34 (0.26–0.44)
	PCB187 (ng/g, serum, lipid-adj.)	MA	Low	275 (8)	1.6 (1.2–2.0)	519 (8)	4.5 (4.1–5.0)	0.35 (0.27–0.45)
	PCB194 (ng/g, serum)	MA	Low	265 (98)	0.0029 (0.0016–0.0052)	508 (53)	0.02 (0.018–0.023)	0.14 (0.079–0.25)
	PCB194 (ng/g, serum, lipid-adj.)	MA	Low	265 (98)	0.50 (0.29–0.87)	508 (53)	3.4 (3.1–3.7)	0.15 (0.085–0.26)
	PCB196 & 203 (ng/g, serum)	MA	Low	274 (63)	0.0038 (0.0025–0.0058)	517 (35)	0.019 (0.017–0.021)	0.20 (0.13–0.31)
	PCB196 & 203 (ng/g, serum, lipid-adj.)	MA	Low	274 (63)	0.65 (0.44–0.97)	517 (35)	3.1 (2.8–3.4)	0.21 (0.14–0.32)
	PCB199 (ng/g, serum)	MA	Low	272 (69)	0.0036 (0.0023–0.0057)	509 (38)	0.021 (0.018–0.023)	0.18 (0.11–0.29)
	PCB199 (ng/g, serum, lipid-adj.)	MA	Low	272 (69)	0.62 (0.39–0.96)	509 (38)	3.4 (3.0–3.8)	0.18 (0.11–0.29)
	PCB206 (ng/g, serum)	MA	Low	271 (41)	0.0041 (0.0029–0.0057)	513 (17)	0.015 (0.013–0.017)	0.27 (0.19–0.39)
	PCB206 (ng/g, serum, lipid-adj.)	MA	Low	271 (41)	0.69 (0.50–0.95)	513 (17)	2.4 (2.2–2.7)	0.28 (0.20–0.39)
	PCB209 (ng/g, serum)	MA	Low	269 (32)	0.0034 (0.0026–0.0044)	510 (17)	0.0092 (0.0078–0.011)	0.37 (0.27–0.51)
	PCB209 (ng/g, serum, lipid-adj.)	MA	Low	269 (32)	0.57 (0.44–0.73)	510 (17)	1.5 (1.3–1.7)	0.38 (0.28–0.51)
Metals (2007–2008)	BARIUM (ug/g, urine, creatinine-adj.)	NHB	High	284 (2)	0.87 (0.79–0.96)	581 (3)	1.9 (1.7–2.1)	0.46 (0.40–0.54)
	BARIUM (ug/g, urine, creatinine-adj.)	NHB	Low	264 (6)	0.84 (0.72–0.99)	581 (3)	1.9 (1.7–2.1)	0.45 (0.36–0.56)
	CESIUM (ug/g, urine, creatinine-adj.)	NHB	High	284 (0)	3.6 (3.4–3.9)	581 (0)	4.8 (4.6–5.1)	0.75 (0.68–0.83)
	CESIUM (ug/g, urine, creatinine-adj.)	NHB	Low	264 (0)	3.2 (3.0–3.4)	581 (0)	4.8 (4.6–5.1)	0.66 (0.59–0.73)
	COBALT (ug/g, urine, creatinine-adj.)	NHB	High	284 (0)	0.30 (0.28–0.32)	581 (2)	0.39 (0.37–0.41)	0.76 (0.71–0.82)
	MERCURY, TOTAL (ug/L, blood)	MA	Low	1,056 (314)	0.53 (0.47–0.59)	1,819 (375)	0.82 (0.73–0.93)	0.65 (0.55–0.76)
	MERCURY, TOTAL (ug/L, blood)	NHW	Low	1,438 (503)	0.48 (0.4–0.59)	1,819 (375)	0.82 (0.73–0.93)	0.59 (0.51–0.69)
Perchlorate (2003–2004)	PERCHLORATE (ug/g, urine, creatinine-adj.)	NHB	High	233 (0)	2.1 (1.7–2.5)	673 (0)	3.7 (3.3–4.1)	0.57 (0.46–0.70)
	PERCHLORATE (ug/g, urine, creatinine-adj.)	NHB	Low	365 (0)	2.5 (2.2–2.8)	673 (0)	3.7 (3.3–4.1)	0.67 (0.61–0.73)
PFCs (2007–2008)	PERFLUOROOCTANE SULFONIC ACID (ug/L, blood)	MA	Low	234 (0)	9.6 (8.4–11)	500 (1)	14 (13–16)	0.67 (0.57–0.78)
	PERFLUOROOCTANOIC ACID (ug/L, blood)	MA	Low	234 (0)	3.3 (3.0–3.6)	500 (0)	4.5 (4.3–4.8)	0.73 (0.66–0.80)
	PERFLUOROOCTANOIC ACID (ug/L, blood)	NHB	Low	204 (1)	3.5 (3.3–3.8)	500 (0)	4.5 (4.3–4.8)	0.78 (0.74–0.83)
Phenols (2007–2008)	BENZOPHENONE-3 (ng/mL, urine)	NHB	High	258 (18)	8.1 (6.1–11)	588 (20)	30 (18–49)	0.27 (0.16–0.45)
	BENZOPHENONE-3 (ng/g, urine, creatinine-adj.)	NHB	High	258 (18)	6.0 (4.6–7.6)	588 (20)	31 (19–52)	0.19 (0.11–0.32)

Notes: (a) Biomarkers were assigned to chemical groups on the basis of chemical consistency and groupings from the NHANES laboratory files. Biomonitoring data for the results in the table come from NHANES 2007–2008 for metals, PFCs, and phenols; and from NHANES 2003–2004 for halogenated aromatics and perchlorate. (b) MA – Mexican American, NHB – non-Hispanic black, NHW – non-Hispanic white; Low income corresponds to family PIR < 2, High income corresponds to family PIR ≥ 2 (PIR is the poverty income ratio). (c) The number of observations is unweighted. The number of non-detects (ND) is reported in parentheses. (d) The point estimate, followed by the range estimate in parentheses. The range estimate is a 95% confidence interval unadjusted for multiple comparisons. GM and GMR estimates are rounded to two significant figures. (e) The reference subgroup was non-Hispanic white/High income.

Of the 12 chemicals our screening method identified as having higher concentrations in low-income or minority groups (Table 1), we found published evidence of EJ concern for cotinine, lead, p,p’-dichlorodiphenyldichloroethylene (DDE), methyl and propyl paraben, phthalates, and antimony (Sb), and no published evidence for thallium (Tl) or dichlorophenols.

### Cotinine

We found income-related disparity in cotinine and other tobacco smoke biomarkers (e.g., Pb and Sb). There is an established literature on higher smoking rates in low-income U.S. subpopulations [33].

### Lead

We found significantly higher blood and urine Pb among low-income, non-Hispanic blacks, despite the fact that blood and urine Pb have been found to be weakly correlated and that blood Pb is considered a more reliable biomarker than urine Pb [34,35]. Our finding agrees with Pirkle et al. [36], who found the covariates non-Hispanic black race and low income to be significantly positively associated with blood Pb across all age groups in a multiple regression analysis, using NHANES 1991–1994 data, and with results reported in Tyrrell et al. [10].

### DDE

We found elevated serum p,p’-DDE—a ubiquitous, neurotoxic dichlorodiphenyltrichloroethane (DDT) metabolite—among Mexican Americans. CDC reported that Mexican Americans had the highest levels in NHANES 1999–2004, with the 2003–2004 GM dropping >30% from earlier cycles, a possible result of Mexico’s 2000 DDT phase-out and the high proportion of new immigrants among Mexican Americans [37]. A study of low-income, pregnant Mexican American women in California found higher p,p’-DDE associated with time spent living outside the United States and with birthplace in areas of Mexico with recent DDT use [38]. We further examined the NHANES 2003–2004 organochlorine pesticide data and found a higher proportion of born-in-Mexico (versus born-in-U.S.) participants in the Mexican American/Low Income subgroup (0.59; 95% CI 0.47–0.72) versus the High Income subgroup (0.37; 95% CI 0.27–0.47), which may help explain our finding.

### Parabens

Similar to the NHANES 2005–2006 findings of Calafat et al. [39], we observed higher urinary methyl and propyl paraben concentrations among high-income, non-Hispanic blacks in NHANES 2007–2008. We also found methyl paraben to be elevated among low-income, non-Hispanic blacks. The methyl paraben result for high-income blacks was not sensitive to whether the measurements were creatinine-corrected, consistent with descriptive statistics reported by CDC for non-Hispanic blacks [9].

### Phthalates

We found higher diethyl phthalate (urinary mono-ethyl phthalate) and dibutyl phthalate (urinary mono-isobutyl and mono-n-butyl phthalate) metabolites in low-income minority subgroups, with higher mono-ethyl phthalate also in high-income, non-Hispanic blacks. Race/ethnicity differences in exposure to these phthalate metabolites were previously documented [40], and evidence regarding income-related differences is conflicting. Higher exposures to summed urinary metabolites of low-molecular-weight phthalates were reported for minority and for lower-income children (ages 6–19) [41]; inverse associations between dibutyl phthalate metabolites and income (controlling for race) were also found in NHANES 2001–2010 [10]. Controlling for SES (an index including income, education, and food security), elevated urinary mono-ethyl phthalate and dibutyl phthalate metabolites were found in minority reproductive-age women, but SES itself was insignificant in the presence of minority status controls [42].

### Sb and Tl

We found higher urinary Sb (uncorrected) in non-Hispanic blacks (low and high income) and higher urinary Tl (uncorrected) in low-income non-Hispanic blacks. Richter et al. [43] found higher urinary Sb in NHANES 1999–2004 non-smokers with environmental tobacco smoke exposure compared to non-smokers with no such exposure, but no difference by race/ethnicity. In contrast, they found lower urinary Tl in smokers versus non-smokers. Tyrrell et al. reported negative associations between Sb and income (controlling for race) [10], but no race-related differences.

### Dichlorophenols

We found elevated 2,4– and 2,5-dichlorophenol (DCP) in non-Hispanic blacks (low and high income). Evidence of EJ concerns exists for the 2,5-DCP parent compound (1,4–dichlorobenzene), with elevated blood levels found in Mexican Americans and non-Hispanic blacks [44,45]. Urinary 2,5–DCP was found to be significantly lower in non-Hispanic white girls compared to non-Hispanic black girls participating in a breast cancer study [46].

### Chemicals with higher concentrations in the reference subgroup

The screening method identified 25 chemicals with significantly higher biomarker levels in high-income, non-Hispanic whites (Table 2), with previously published evidence of disparity for most. We found lower serum levels of 17 PCB congeners in Mexican Americans than in high-income, non-Hispanic whites, consistent with other NHANES studies [47,48] and regional U.S. studies [49]. All of the congeners with significant differences, except PCB118 and PCB156, were non-dioxin like, and results were not sensitive to the lipid adjustment. We found lower total blood mercury (Hg) in low-income, non-Hispanic whites and Mexican Americans, consistent with studies reporting lower Hg levels among Mexican Americans [50,51] and inverse associations between Hg and income [10]. We found lower urinary perchlorate in non-Hispanic blacks (low- and high-income) versus high-income, non-Hispanic whites in NHANES 2003–2004, similar to Blount et al.’s [52] NHANES 2001–2002 finding for men. We found lower serum perfluoroctanoic acid (PFOA) in the low-income minority subgroups and lower perfluorooctane sulfonic acid (PFOS) among low-income Mexican Americans, consistent with NHANES 1999–2008 findings of lower PFOA and PFOS in Mexican Americans and lower PFOA in blacks [53], and with NHANES 2003–2008 findings of negative associations between income and PFOA and PFOS levels [10,54]. Lower urinary benzophenone-3 was found in blacks in NHANES 2003–2004 [55] and higher-income individuals in NHANES 2003–2010 [10], similar to our NHANES 2007–2008 finding in high-income, non-Hispanic blacks. Last, we found lower concentrations of urinary barium (Ba) and cesium (Cs) in non-Hispanic blacks (low- and high-income), and lower concentrations of cobalt (Co) in high-income, non-Hispanic blacks, compared to high-income, non-Hispanic whites. Although there is evidence of inverse associations between Cs and income (controlling for race) in NHANES 2003–2007 [10], we did not locate any published evidence of race/ethnicity-related disparities in these metals.

## Discussion

### Utility and performance of the screening method

This analysis provides a formal method to screen for exposure disparities in NHANES environmental biomonitoring data across race/ethnicity and income. The screening method identified differential exposure at the mean for 59 of the 204 (29%) biomarkers examined, with some instances of potential EJ concern and others where the reference subgroup (non-Hispanic white/High Income) had higher exposures. Using the published literature as a qualitative validation tool, the method correctly identified five chemicals/chemical classes with published evidence of higher biomarker levels in low-income or minority groups (cotinine, lead, DDE, parabens, and phthalates), and five chemicals/chemical classes with higher levels in high-income whites (PCBs, Hg, perchlorate, PFOA/PFOS, and benzophenone-3). It also found differential exposures for seven chemicals (2,4- and 2,5-DCP, Tl, Sb, Ba, Co, Cs) for which no published evidence of differences by race/ethnicity or income exists. The screening method is an approach that users of NHANES biomonitoring data could employ to obtain new and robust insights into the nexus between chemical exposures and diverse populations.

### Public health relevance of initial screening results

The main objective of this work was to develop an EJ screening method for the NHANES biomarker data. Because we used only one cycle of NHANES data to develop the method (the most recently available per chemical), our actual screening results should be viewed as preliminary. Furthermore, there are many other potentially important race/ethnicity disparities that we were unable to evaluate because the NHANES dataset contains sufficient sample sizes to reliably analyze only Mexican American, non-Hispanic white, and non-Hispanic black subgroups [16]. Nonetheless, we did find evidence, supported by the published literature, of EJ concern in biomarker levels of cotinine, Pb, DDE, parabens, and phthalates. While smoking is not typically viewed as an EJ issue, higher cotinine levels in low-income, non-Hispanic whites and blacks indicate higher smoking-related health burdens. This merits further investigation into factors driving higher smoking rates and secondhand smoke exposures, so that effective prevention strategies can be developed. We also found higher Pb in low-income blacks. An extensive literature points to indoor/housing-related factors (e.g., house dust, tobacco smoke, housing age/condition/geographic location) as important drivers of Pb exposure in the United States, with dietary, toxicokinetic, and genetic factors influencing biomarker differences [56]. With research demonstrating adverse effects at ever-decreasing Pb levels, including associations with cardiovascular outcomes [35,57], the public health impacts of Pb disparities are potentially large.

We found higher DDE levels among Mexican Americans. Since prenatal p,p’-DDE exposure is associated with adverse neurodevelopmental outcomes [37], the public health impacts of this disparity could be significant. We found higher paraben levels among high-income, non-Hispanic blacks. Parabens are antimicrobial preservatives with weak estrogenic properties used in cosmetics, pharmaceuticals, and some processed foods [58]. Exposure differences are likely due to product use or diet, although indoor air and house dust may be important [59]. We found certain phthalate metabolites higher in low-income minority subgroups and high-income, non-Hispanic blacks. Phthalates are ubiquitous plasticizers, with diet and consumer products considered important exposure sources [6]. Human health implications of phthalate exposure is an active research area, with some suggestion of endocrine-disrupting effects [41,60].

We also found previously undocumented evidence of disparities in biomarker levels of Sb, Tl, and 2,4- and 2,5-DCP for non-Hispanic blacks. Sb and Tl are toxic metals used in a range of industrial processes. Anthropogenic sources include power plants (both), traffic emissions and brake dust (Sb), tobacco smoke (both), mining operations (Sb), cement factories and smelters (Tl), and waste sites (both) [61]. People are exposed to Sb primarily through food and to Tl through industrial processes [6]. Human health effects from Sb or Tl at low environmental doses are unknown [6]. 2,4–DCP is a metabolite of several herbicides, organophosphate, and organochlorine pesticides (including other chlorophenols), while 2,5–DCP is a metabolite of several organochlorine pesticides (including 1,4–dichlorobenzene, a deodorizer and moth repellent) [6,62,63]. They can also be used in water chlorination [64]. In terms of potential health effects, food allergy sensitization was more common in NHANES 2005–2006 participants with levels of urinary 2,4– and 2,5–DCP above the 75th percentile [64], and lower age of menarche was associated with 2,5–DCP and aggregated 2,4– and 2,5–DCP in NHANES 2003–2008 female participants 12–16 years of age [65].

We also found evidence, supported by the literature, of lower levels of certain chemicals in low-income and minority subgroups versus high-income non-Hispanic whites. PCB levels were lower in Mexican Americans, most likely due to differences in diet, the younger average age of Mexican Americans (34 years; 95% CI 31–36) versus whites (44 years; 95% CI 43–45), and the large fraction (0.56; 95% CI 0.42–0.66) of Mexican-born participants (who have been shown to have lower levels of PCB153 than U.S.-born Mexican Americans [48]) in the Mexican American subgroups in the NHANES 2003–2004 data. Hg levels were lower in low-income non-Hispanic whites and Mexican Americans, consistent with studies linking higher income with higher Hg intake through fish consumption [6,10,50,51]. We found lower perchlorate in non-Hispanic blacks, and lower PFOA and PFOS in the low-income minority subgroups. Perchlorate is a thyrotoxic natural and anthropogenic contaminant found in food (vegetables, milk) and drinking water, depending on location. PFOA and PFOS (phased out of U.S. production in 2002) are persistent manmade chemicals with a range of applications (e.g., waterproofing, protective coatings) and suspected health effects. Levels of benzophenone-3, a suspected endocrine-disrupting sunscreen used in cosmetics, sunscreen, and food packaging, were lower in high-income non-Hispanic blacks.

Last, we found previously undocumented evidence of lower Ba, Cs, and Co in non-Hispanic blacks compared to high-income non-Hispanic whites. Ba is a naturally occurring metal in food and drinking water, with industrial and medical applications [66]; disparities could indicate differences in diet, drinking water, or possibly access to colorectal screening. Americans are exposed to both stable (naturally occurring, and from forest fires, coal, and waste combustion; not considered a public health concern) and radioactive (from nuclear power plants, accidents, or weapon explosions) Cs isotopes through food, drinking water, and air; thus, disparities are likely due to differences in diet and geography [67]. Americans are also exposed to stable and radioactive Co isotopes through food, water, and air. Since Co is an essential micronutrient, exposure to typical environmental levels of stable Co is not considered harmful [68]. Urinary Cs and Co measurement methods do not distinguish between stable and radioactive species.

### Limitations

We were unable to analyze 50% of the available NHANES biomarkers for disparity because the LOD censoring was often too high to yield a valid GM estimate. In the VOC chemical group this was true for 33 out of 39 biomarkers. However, the lack of information about race/ethnicity and income differences for these biomarkers should not be interpreted as the absence of such differences. With improvements in the sensitivity of analytical methods, LOD censoring should become less of a limitation.

For 71% of biomarkers, none of the estimated GMRs was significantly different from 1. This was the case for all 20 PAH biomarkers. The lack of significant findings for these biomarkers may be a consequence of insufficient statistical power; in other words, race/ethnicity and income differences may exist, but we were unable to detect them. Pooling biomarker data across several NHANES cycles would have increased sample sizes and, potentially, the precision of our estimates, but would not have altered our conclusions about the validity of the screening method itself.

To control the FWER in the family of 795 screening tests performed, we used the Holm-Bonferroni procedure. While this procedure was shown to be more powerful than the Bonferroni correction [30], it does not permit construction of simultaneous confidence intervals. Therefore, the confidence intervals for the GMRs reported in Table 1 and Table 2 are not adjusted for multiple comparisons. There are several other FWER control methods that have higher statistical power compared to the Holm-Bonferroni procedure. Adaptive Bonferroni methods require knowledge of (or assumptions about) data dependencies [69,70]. However, we could not infer the correlation structure across all NHANES biomarkers, because not all measurements were collected from the same individuals. Permutation-based methods, such as the MaxT test procedure [71], accommodate arbitrary dependency structures. However, they rely on the assumption that individual-level observations are exchangeable, which is difficult to justify for the complex survey data. Further, the MaxT procedure did not considerably outperform the Holm-Bonferroni procedure in terms of statistical power in some simulation experiments [72,73]. Thus, we used an approach to control the FWER that we felt was most appropriate for these data.

There are other types of disparity that we were unable to capture by screening at the means. Higher variability in a given biomarker concentration in a target subgroup (versus the reference subgroup) implies that, even with similar GMs, extreme values may be more frequent in that subgroup. We explored this in a complementary, upper-tail-oriented screening that defined extreme concentrations (as ≥95th percentile) and found few significant results. This was likely a consequence of the additional sampling uncertainty in the test statistic estimator used for this upper-tail screening, because the 95th percentile value had to be estimated from the data. When juxtaposed with results from screening at the mean, fewer significant findings at the upper tail could be misinterpreted as a relative absence of the upper-tail disparity. Therefore, we did not report the results of this analysis.

Ideally, an upper-tail screening analysis would be based on externally defined, non-occupational health-based thresholds, such as biomonitoring equivalents (BEs). A BE is a biological concentration of a chemical (or its metabolites) reflecting an existing health-based exposure guidance value, such as a reference dose [74]. BEs have been established for approximately 80 chemicals [74], but many NHANES chemicals still lack them. Additionally, grouping biomarkers of chemicals with shared toxicity pathways could help capture toxicity-relevant race/ethnicity and income differences in cumulative exposures.

Education and occupation are other important SES dimensions we did not examine explicitly because we analyzed biomarker data for all available ages, where these are not always applicable. For adults, education is typically correlated with income; thus, our income-related findings could be viewed more broadly as representing income- and education-related patterns. However, another study found that, while education and income were correlated, they were not associated with bisphenol A and PFC biomonitoring levels in the same way [54]. Some of the NHANES biomarker concentrations may have reflected occupational exposures, which may occur more frequently in low-income subpopulations (and for some race/ethnicity subpopulations). However, if one assumes that worker exposures are higher than those of the general population, then our focus on disparities at the mean likely helped dampen the influence of occupation on our screening findings. Future detailed studies should consider occupation as an important potential source of variability in biomarker data and possible explanation for observed disparities. Unfortunately, the NHANES occupational codes typically do not contain the detail needed to identify specific high-exposure industries or job tasks.

Our screening analysis did not capture possible “hotspot” effects, such as elevated biomarker levels in communities near contaminant sources. Community-level occurrences of elevated concentrations are either diluted or missed altogether if these communities are not included in the NHANES sample. Although the NHANES geographic identifiers are available through special arrangement, accessing them requires additional time and resources; only a few researchers to date have attempted this [75]. Further, having the geographic identifiers alone cannot help elucidate whether biomarker disparities are due to environmental contamination without the corresponding local environmental data (e.g., air, drinking water, soil, house dust, food measurements), which NHANES generally lacks. For this, we need more detailed studies, such as those described in the introduction [3,4], matching environmental, geographic, and SES data.

Interpreting urinary biomarker levels when results differ by creatinine correction can be challenging. Because non-Hispanic blacks have higher creatinine excretion [15], GMRs that were not significant for creatinine-corrected concentration (but significant for uncorrected concentration) may reflect creatinine excretion rather than exposure differences. However, creatinine also varies by several other factors (e.g., age, sex, renal function, lean muscle mass, red meat consumption [15]). We did not account for these factors in our analysis, clouding the interpretation of different results for urinary concentrations expressed in different units. Other approaches to account for urine dilution (e.g., by specific gravity [76]) may be preferable when 24-hour samples are unavailable.

Other limitations of our analysis relate to the inherent characteristics of a screening-level exploration versus a detailed EJ analysis of the NHANES biomonitoring data. Several studies focusing on clusters of a few chemicals have demonstrated the value of individual-level covariates—such as age, sex, education, occupation, smoking, diet, and body mass index—in explaining biomarker differences across EJ subgroups. However, the set of important covariates could also include genetic/epigenetic characteristics that influence toxicokinetics, resulting in different internal doses for individuals with the same external exposures [7,8]. Although NHANES is a rich source of individual-level information, it does not provide genetic/epigenetic data.

This screening analysis focused on identifying race/ethnicity and income differences in mean concentrations for a large number of the NHANES biomarkers, rather than on interpreting these differences. Making inferences about factors that can account for these observed differences should be assisted by a correctly specified model of individual-level internal exposures that includes all relevant covariates. It was not feasible to build a comprehensive model for each biomarker in our study. Further, including just a few covariates (e.g., age and sex) was likely to produce models subject to omitted-variable bias and, consequently, faulty inferences about the relative importance of these covariates in explaining the mean differences in exposure across subgroups. Therefore, we focused on a simpler screening that could potentially be useful for identifying candidate chemicals for more detailed EJ-oriented assessments.

## Conclusions

This analysis explored differences in exposure to environmental chemicals (using biomarkers) across the dimensions of race/ethnicity and income in the United States. Many findings were consistent with previous studies, while some findings were new. Screening analyses of this type can be useful in identifying chemicals for focused study. Researchers wishing to extend our analyses might consider upper-tail screening using BE-based thresholds, exploring patterns in cumulative exposure (by grouping biomarkers with shared toxicity), or examining effects of creatinine correction and lipid adjustment on findings for certain chemical groups. Incorporating additional years of NHANES data as they become available could help identify persistent disparities requiring public health attention.

## Abbreviations

ATSDR: Agency for toxic substances and disease registry; Ba: Barium; BE: Biomonitoring equivalent; CDC: Centers for disease control and prevention; CI: Confidence interval; Co: Cobalt; Cs: Cesium; DCP: Dichlorophenol; DDE: Dichlorodiphenyldichloroethylene; DDT: Dichlorodiphenyltrichloroethane; EJ: Environmental justice; EPA: U.S. Environmental protection agency; FWER: Family-wise error rate; GM: Geometric mean; GMR: Geometric mean ratio; GSD: Geometric standard deviation; Hg: Mercury; LOD: Limit of detection; NHANES: National health and nutrition examination survey; PAH: Polycyclic aromatic hydrocarbon; Pb: Lead; PCB: Polychlorinated biphenyl; PFC: Polyfluoralkyl chemicals; PFOA: Perfluoroctanoic acid; PFOS: Perfluorooctane sulfonic acid; PIR: Poverty income ratio; Sb: Antimony; SES: Socioeconomic status; Tl: Thallium; VOCs: Volatile organic compounds.

## Competing interests

The authors declare they have no competing interests. Although the research described in this article has been funded in part by the U.S. Environmental Protection Agency (EPA) contracts EP-W-05-022 and EP-W-06-044 to Abt Associates Inc., it has not been subject to the Agency’s review and therefore does not necessarily reflect the views of the Agency, and no official endorsement should be inferred.

## Authors’ contributions

MC conceived of and guided the analysis. AB, SLG, and LO developed the screening method. AB implemented the data analysis. AB, SLG, and AMR drafted the manuscript. All authors contributed to, read, and approved the final version of the manuscript.

## Supplementary Material

Additional file 1 **A Method to screen U.S. environmental biomonitoring data for race/ethnicity and income-related disparity.** A description of chemical groups and their corresponding NHANES laboratory files and an overview of significant GMR findings. Click here for file
