# Supplementary material for: A Method to screen U.S. environmental biomonitoring data for race/ethnicity and income-related disparity
Source: Environ Health. 2013 Dec 19;12:114. doi: 10.1186/1476-069X-12-114 (PMC3893603; doi:10.1186/1476-069X-12-114)
Supplement: Additional file 1 — A Method to screen U.S. environmental biomonitoring data for race/ethnicity and income-related disparity. A description of chemical groups and their corresponding NHANES laboratory files and an overview of significant GMR findings. [file 1476-069X-12-114-S1.docx]

# Supporting information

# A Method to screen U.S. environmental biomonitoring data for race/ethnicity and income-related disparity

Anna Belova, Susan L. Greco, Anne M. Riederer, Lauren E.W. Olsho, Mark A. Corrales

CONTENTS

Table A1 - Chemical groups and their corresponding NHANES laboratory files

Table A2 - Overview of significant GMR findings

## Table A1 - Chemical groups and their corresponding NHANES laboratory files

| **Chemical Group^a^** | **NHANES Laboratory Data File** | **NHANES Cycle** | **Number of  Biomarkers** |
| --- | --- | --- | --- |
| Cotinine | Lab06 Serum Cotinine | 2007-2008 | 3 |
| Halogenated Aromatics | Lab28 Dioxins Furans and Coplanar PCBs | 2003-2004 | 58 |
|  | Lab28 NonDioxin Like PCBs | 2003-2004 | 52 |
|  | Lab28 Polybrominated Diphenyl Ethers | 2003-2004 | 22 |
| Metals | Lab06 Blood Cadmium and Lead | 2007-2008 | 2 |
|  | Lab06 Blood Total Mercury and Blood Inorganic Mercury | 2007-2008 | 2 |
|  | Lab06 Urinary Heavy Metals | 2007-2008 | 24 |
|  | Lab06 Urinary Mercury | 2007-2008 | 2 |
|  | Lab06 Urinary Total and Speciated Arsenic | 2007-2008 | 16 |
| PAHs | Lab31 Polyaromatic Hydrocarbons | 2003-2004 | 20 |
| Perchlorate | Lab04 Urinary Perchlorate | 2003-2004 | 2 |
| Pesticides | Lab24 Environmental Pesticides | 2007-2008 | 10 |
|  | Lab26 Pesticides | 2003-2004^b^ | 68 |
|  | Lab26 Urinary Organophosphate Insecticides | 2003-2004 | 12 |
|  | Lab28 Organochlorine Pesticides | 2003-2004 | 26 |
| PFCs | Lab24 Polyfluorinated Compounds | 2007-2008 | 12 |
| Phthalates | Lab24 Urinary Phthalates | 2007-2008 | 30 |
| Phenols | Lab24 Environmental Phenols | 2007-2008 | 16 |
| VOCs | Lab04 Volatile Organic Compounds | 2003-2004 | 33 |
| Notes: (a) Biomarkers were assigned to chemical groups on the basis of groupings from the NHANES laboratory files and chemical consistency. (b) One chemical was taken from the 2001-2002 cycle: 3,5,6-TRICHLOROPYRIDINOL | | | |

## Table A2 - Overview of significant GMR findings

| **Reported Quantity** | **Level of reporting^a^** | | |
| --- | --- | --- | --- |
|  | **Comparisons** | **Biomarkers** | **Chemicals** |
| All Potential | 2050 | 410 | 228 |
| Calculated GMR^b^ | 795 | 204 | 108 |
| GMR^c^ statistically different from 1 | 86 | 59 | 37 |
| **GMR significantly greater than 1** | **31** | **16** | **12** |
| **GMR significantly less than 1** | **55** | **43** | **25** |
| Notes: GMR is the geometric mean ratio. (a) For each biomarker, up to five comparisons can be made to the reference subgroup using the GMR. In this table, we report the number of biomarkers with at least one GMR satisfying the reported quantity condition. One chemical could be measured using several biomarkers. (b) Not all GMRs could be calculated due to data limitations. (GM not reported when >40% of concentrations are below the LOD). (c) The statistical significance level was 5%. The Holm-Bonferroni procedure was used to control for multiple comparisons. | | | |

Table A2 summarizes the overall GMR screening results. One biomarker could be measured in different media or using different corrections (e.g., blood lead (Pb); urinary Pb—unadjusted and creatinine-corrected). We considered a total of 410 biomarkers in our analyses. For each, up to five GMR tests could be performed comparing each target subgroup to the reference. A comparison was possible only if the GMR could be estimated. Of 2050 potential comparisons, we could make 795, since large fractions of <LOD values did not allow GM estimates for many subgroups. Of the 795, there were 86 (11%) in which the null hypothesis (GMR=1) was rejected: 31 cases demonstrated EJ concerns with the subgroup GM significantly exceeding that of the reference.

We aggregated results by counting the number of biomarkers for which at least one comparison could be made, at least one null hypothesis could be rejected, and at least one GMR was significantly >1. There were 204 biomarkers with at least one GMR estimated, 59 (29%) with evidence of disparity, and 16 with at least one GMR significantly >1. At the chemical level, 37 chemicals showed evidence of disparity with 12 having at least one GMR significantly >1.
